# A framework for clinical commissioning of 3D‐printed patient support or immobilization devices in photon radiotherapy

**DOI:** 10.1002/acm2.12408

**Published:** 2018-07-08

**Authors:** Tyler Meyer, Sarah Quirk, Malgorzata D'Souza, David Spencer, Michael Roumeliotis

**Affiliations:** ^1^ Department of Oncology University of Calgary Calgary AB Canada; ^2^ Department of Physics and Astronomy University of Calgary Calgary AB Canada; ^3^ Medical Physics Department Tom Baker Cancer Centre Calgary AB Canada

**Keywords:** 3D printing, clinical implementation, commissioning, immobilization, megavoltage

## Abstract

**Purpose:**

The objective of this work is to outline a framework for dosimetric characterization that will comprehensively detail the clinical commissioning steps for 3D‐printed materials applied as patient support or immobilization devices in photon radiotherapy. The complex nature of 3D‐printed materials with application to patient‐specific configurations requires careful consideration. The framework presented is generalizable to any 3D‐printed object where the infill and shell combinations are unknown.

**Methods:**

A representative cylinder and wedge were used as test objects to characterize devices that may be printed of unknown, patient‐specific dimensions. A case study of a 3D‐printed CSI immobilization board was presented as an example of an object of known, but adaptable dimensions and proprietary material composition. A series of measurements were performed to characterize the material's kV radiologic properties, MV attenuation measurements and calculations, energy spectrum water equivalency, and surface dose measurements. These measurements complement the recommendations of the AAPM's TG176 to characterize the additional complexity of 3D‐printed materials for use in a clinical radiotherapy environment.

**Results:**

The dosimetric characterization of 3D‐printed test objects and a case study device informed the development of a step‐by‐step template that can easily be followed by clinicians to accurately and safely utilize 3D‐printed materials as patient‐specific support or immobilization devices.

**Conclusions:**

A series of steps is outlined to provide a formulaic approach to clinically commission 3D‐printed materials that may possess varying material composition, infill patterns, and patient‐specific dimensions.

## 1 INTRODUCTION

Three‐dimensional (3D) printing technology has substantially improved over the past decade and is already being used within radiotherapy for a variety of applications, including bolus,^1^, ^2^, ^3^ brachytherapy applicators,^4^ quality control and anthropomorphic phantoms,^5^, ^6^, ^7^, ^8^ and preclinical immobilization.^2^, ^9^ Technology variations across 3D printer vendors introduce different material compositions and print infill patterns that are often proprietary and possess unknown radiologic properties for use in radiotherapy. This contrasts with most commercially manufactured immobilization devices specifically designed for radiotherapy where it is in a vendor's best interest to provide radiotherapy departments with detailed device specifications.

The AAPM's TG176 reported on dosimetric effects caused by couch tops and immobilization devices.^10^ The manuscript included a comprehensive list of existing literature that characterizes the dosimetric properties of common, commercial radiotherapy devices. With a 3D printer, objects can be individually customized by the clinic to accommodate a specific patient's treatment and, therefore, it is not feasible to comprehensively characterize every device, material, and geometry configuration that may be manufactured for patient use. This is especially true with 3D printing applications designed for patient specific anatomy, which may require printed devices that possess patient‐specific configurable arrangements or are disposable. For example, characterization of a 3D‐printed device's attenuation at varying beam obliquity is not practical when a device's thickness is not known prior to modifying the design for patient specific anatomy.

Additionally, TG176 primarily characterizes patient support and immobilization devices through attenuation and surface dose effects. TG176 serves as the foundation for understanding the dosimetric impact of traditional radiotherapy devices, but additional information is required for 3D‐printed materials that is dependent on proprietary material composition and print infill pattern.

In this manuscript, a framework is provided for dosimetric characterization of 3D‐printed materials that may be utilized without fixed device dimensions. This work builds upon the recommendations of AAPM's TG176 to include additional characterization techniques specifically for 3D‐printed materials. An immobilization device to be used clinically in volumetric‐modulated arc therapy (VMAT) cranio‐spinal irradiation (CSI) is used as a case study to demonstrate the results of the outlined methodology. The characterization experiments that align with AAPM's TG176 are reported in this work in the context of performing the measurements on the sample 3D‐printed device. As well, the additional characterization measurements outlined specifically for 3D‐printed materials are described in detail.

## 2 METHODS

All measurements were performed with a proprietary material (Onyx^™^) that was printed from the Markforged Onyx One (Markforged, Boston, MA, USA). Onyx^™^ is a carbon fiber‐based material with a nylon additive, of proprietary composition. The 3D‐printed objects used in the following experiments were printed with a nominal 50% infill and 1‐mm shell thickness. The framework presented is applicable to any 3D‐printed object where the infill and shell combinations are unknown. All linear accelerator‐based measurements were performed on Varian accelerators (Varian Medical Systems Inc., Palo Alto, CA, USA).

### 2.A Representative 3D‐printed material

**Figure 1:**
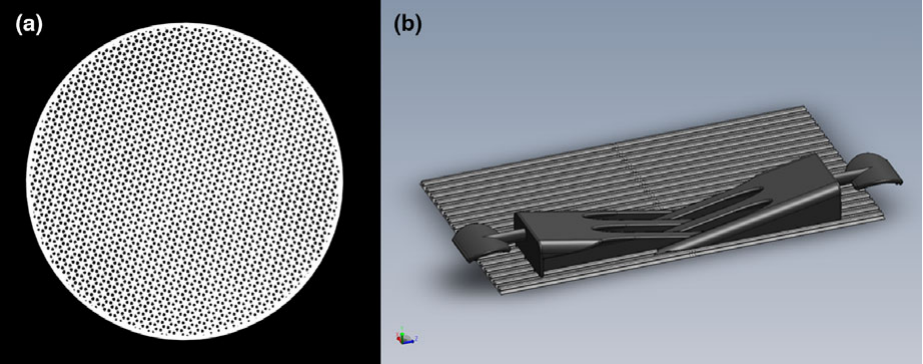
3D‐printed devices used for radiologic characterization. (a) A coronal slice of a CT image of the representative cylinder, and (b) 3D rendering of the CSI immobilization board to be used clinically.

To characterize the 3D‐printed material for general use in radiotherapy, two representative test objects were used: a cylinder [Fig. 1(a)] was printed with 15‐cm diameter and 3‐cm height and a wedge (not shown) with 3‐cm height and 10‐cm length. The representative cylinder diameter was selected so that a reference 10 × 10 cm^2^ field would fit entirely over the diameter of the device. The cylinder height was selected so that the ratio of infill pattern to shell thickness is clinically similar to the intended use of the clinical immobilization device. The representative wedge height was selected to match the height of the cylinder. The density of the representative cylinder was estimated to be 0.456 g/cm^3^, which is the quantity used to estimate the water equivalent thickness for experimental analysis.

### 2.B Case Study: VMAT CSI immobilization board

This CSI immobilization board [Fig. 1(b)] has been designed so that it is highly adaptable by easily movable sets of interlocking wedges along a thin 3D‐printed grooved surface. The movability of the wedges allows the same immobilization device to be used for the smallest pediatric patients and the largest adult patients undergoing cranio‐spinal irradiation (CSI) using a VMAT‐based technique. For these experiments, the CSI immobilization board was setup in a configuration representative of its clinical use.

A CT image of the representative cylinder and a 3D rendering of the CSI immobilization board (i.e., the Onyx^™^ devices) are shown in Fig. 1.

### 2.C Determination of kV properties

The representative cylinder was used to assess kilovoltage (kV) radiologic properties of Onyx^™^. The cylinder was imaged at both a high CT resolution and clinical CT resolution on a Philips Big Bore CT scanner (Philips, Amsterdam, the Netherlands). Average Hounsfield Unit (HU) measurements were determined by creating a region of interest (ROI) within the material and reporting the mean and standard deviation of the HU. The CT image parameters for both the high and clinical resolutions are reported in Table 1.

**Table 1:** CT imaging parameters for high and clinical‐resolution images

CT Parameter	High resolution	Clinical resolution	
		Adult	Pediatric
kV	120	120	120
mAs	400	300	180
Slice thickness (mm)	1	2	2
FOV (mm)	72	600	600

### 2.D Attenuation comparison to treatment planning system

**Figure 2:**
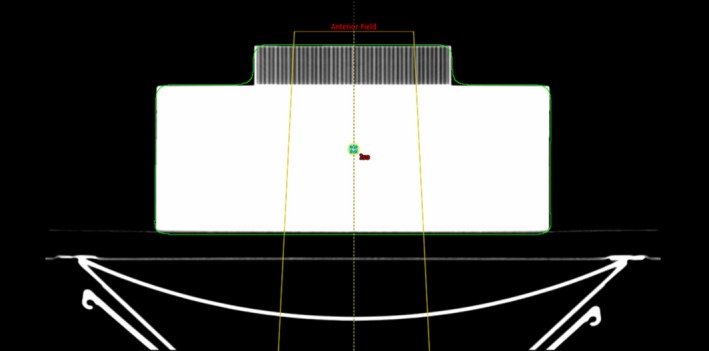
Experimental setup for attenuation measurements. A CT image with SolidWater^®^ and the representative cylinder are shown. Isocenter and the prescription point are located 5 cm depth in SolidWater^®^.

To characterize attenuation of the Onyx^™^ material for the representative cylinder and CSI immobilization board, three experimental setups were used. First, 10 cm of SolidWater^®^ (Best Medical, Springfield, VA, USA) with a Capintec Farmer ionization chamber (Radiation Products Design, Inc, Albertville, MN, USA) placed at 5‐cm depth within the SolidWater^®^ was aligned to the beam's central axis. The surface of the SolidWater^®^ was aligned to 95‐cm source–surface distance (SSD). A 10 × 10 cm^2^ field was used to deliver 100 monitor units (MUs) for all three beam energies that have been clinically commissioned for VMAT CSI: 6 MV, 6 flattening filter free (FFF), and 10 FFF. The same measurement was repeated for all beam energies with the representative cylinder placed atop the SolidWater^®^ and again with the cylinder removed and replaced by the CSI immobilization board. A CT image of the experimental setup described with cylinder atop SolidWater^®^ is shown in Fig. 2.

The ratio of charge collected with and without the cylinder was computed to report attenuation per centimeter of Onyx^™^. The ratio of charge collected with and without the CSI immobilization device was also reported to determine the CSI immobilization board attenuation in a representative treatment arrangement.

A CT image of the three experimental setups used to measure Onyx^™^ attenuation was acquired. The CT images were imported to Eclipse version 13.6 (Varian Medical Systems, Palo Alto, CA, USA) where the experimental setup could be recreated within the treatment planning system. An anterior field was created with the field isocenter and treatment plan's prescription point set to 5‐cm depth (i.e., the ionization chamber location). A prescription dose of 1000 cGy was assigned to the prescription point and dose was calculated with Analytical Anisotropic Algorithm (version 13.6.23, Varian Eclipse). The ratio of monitor units (MUs) with and without the representative cylinder as well as with and without CSI immobilization board was computed.

The ratio of the MUs required to deliver the prescription dose is compared to the ratio of the charge collected by the experimental ionization chamber experiment with the same setup. These results are used to assess the clinical treatment planning system's dose calculation accuracy when the 3D‐printed material is in the beam's path.

### 2.E Energy spectrum water equivalency

Reference percent depth dose (PDD) curves were acquired using a 3D Scanner^™^ (Sun Nuclear, Melbourne, FL, USA) automatic scanning water tank with 10 × 10 cm^2^ field size for 6 MV, 6 FFF, and 10 FFF. The PDDs were acquired from the surface of the water to 30‐cm depth at an ionization chamber scan speed of 0.25 cm/s. For all energies, a second PDD was acquired with the representative cylinder placed at the water surface, termed the Onyx‐based PDD, resulting in a physical SSD of 97 cm. An effective SSD was calculated using the water equivalent thickness of Onyx. Water equivalent thickness was determined by multiplying the physical density and the cylinder thickness (3 cm). The physical density of the representative cylinder was estimated by dividing the measured mass by the calculated cylinder volume.

To evaluate water equivalence for a given energy, the Onyx‐based PDD was compared to the reference PDD. Due to the difference in effective SSDs, the Onyx‐based PDDs were shifted according to an estimate of the water equivalent thickness (1.368 cm) and corrected for inverse square law (ISL).

### 2.F Surface dose measurements

Surface dose measurements were performed separately with the CSI immobilization board, representative cylinder, and representative wedge. All surface dose measurements were performed using GAFchromic^™^ EBT‐3 film (Ashland, Bridgewater, NJ, USA).

The CSI immobilization board experimental setup was selected to compare the intended use of the device to the current clinical standard for treatment. In this application of the CSI immobilization board, the alternative treatment option is having the patient setup directly on the Varian Exact© IGRT couch (Varian Medical Systems, Inc., Palo Alto, CA, USA). Accordingly, surface dose and buildup measurements were compared between the two clinical treatment scenarios.

**Figure 3:**
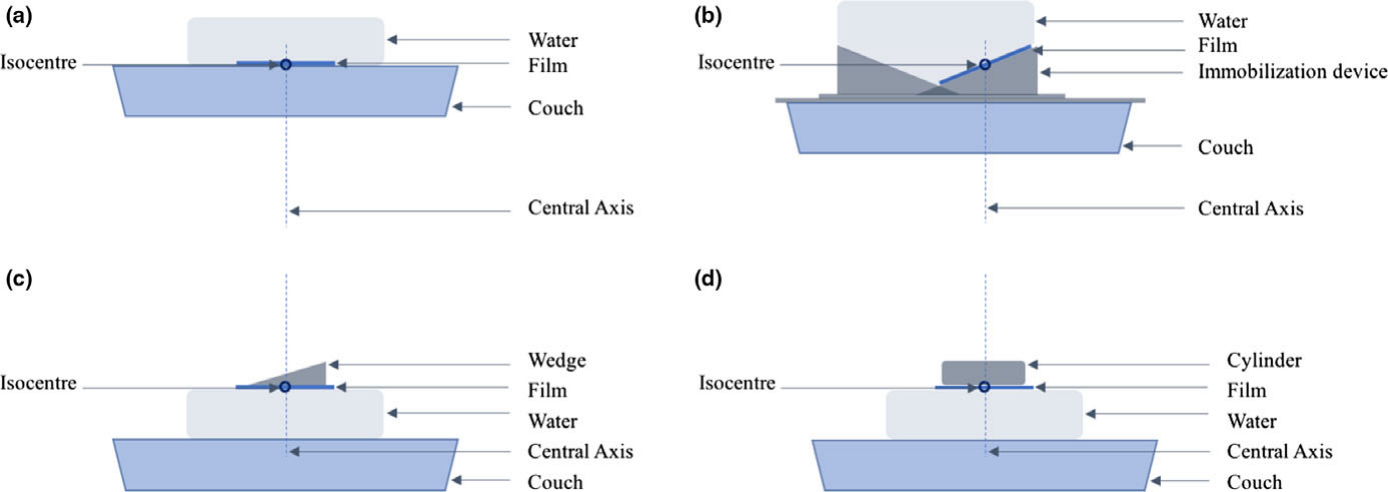
Surface dose measurements using GAFchromic^™^ film. (a) film directly on couch, and b) film on CSI immobilization board, and (c) film beneath the representative wedge, and (d) film beneath the representative cylinder. In all scenarios, the film is placed at isocenter and a 10 × 10 cm^2^ field is delivered with 300 MUs. In (a) and (b), the gantry angle is 180 degrees while in (c) and (d), the gantry angle is 0°.

A film strip was placed directly on the treatment couch with water atop the film to provide backscatter. The center of the film surface was at isocenter (100 cm source‐axis distance). This scenario represents the treatment with patient lying directly on the treatment couch. A similar experiment was conducted with the CSI immobilization board in its expected treatment position on the couch. A film strip was placed directly on the CSI immobilization board on a slat where the patient's back is expected to contact. The center of the film strip was aligned to isocenter. Water was placed atop the CSI immobilization device and film strip. In both sets of measurements, a 6‐MV posterior beam delivered 300 MUs in a reference 10 × 10 cm^2^ field size. The two experimental setups are shown in Figs. 3(a) and 3(b).

To inform the clinician of a surface dose that may be expected if the thickness of the clinical device is varying on a patient‐specific basis, an additional experiment was performed using the representative wedge and cylinder. This experimental setup will allow the clinician to observe the effect of the infill pattern and density on the surface dose. For this experiment, the representative wedge is placed atop film that rests directly on 10 cm of SolidWater^®^. The surface of the SolidWater^®^ is aligned to 100 cm SSD and the film was irradiated by an anterior field with 300 MU. The field size was set such that the width was contained within the width of the wedge and the field length extending beyond the heel and toe of the wedge. The same field size was maintained for the representative cylinder irradiation to directly compare surface dose contributions. Both the wedge and cylinder setup are shown in Figs. 3(c) and 3(d).

The films were calibrated using the methodology outlined by Morrison et al.^11^ To characterize the relative change in surface dose between the two scenarios, films profiles were analyzed in Matlab (Mathworks, Natick, MA, USA).

## 3 RESULTS

### 3.A Determination of kV properties

The ROI measurements on the CT image of the representative cylinder yield a mean (standard deviation) HU of −637 (11.7) and −606 (3.4) for the high resolution and clinical resolution CT images, respectively. A narrower range of HU on the clinical RT resolution is expected due to volume averaging in the CT image.

### 3.B Attenuation comparison to treatment planning system

The measured and calculated dose for the representative cylinder and CSI immobilization board are reported in Table 2. The attenuation per centimeter is reported for the representative cylinder as it provides a measure of the attenuation that could be expected for a device with variable thickness. The total attenuation for a single point measurement is reported for the CSI immobilization board.

**Table 2:** Relative dosimetric difference in Onyx^™^ attenuation. A comparison between measurement and calculation for the representative cylinder and CSI immobilization board

Beam energy	Representative cylinder (attenuation percent per cm)		CSI immobilization board (total attenuation percent)	
	Measurement (%)	Calculation (%)	Measurement (%)	Calculation (%)
6 MV	1.39	1.29	7.76	7.38
6 FFF	1.66	1.51	8.97	8.86
10 FFF	1.31	1.15	6.60	6.68

### 3.C Energy spectrum water equivalency

**Figure 4:**
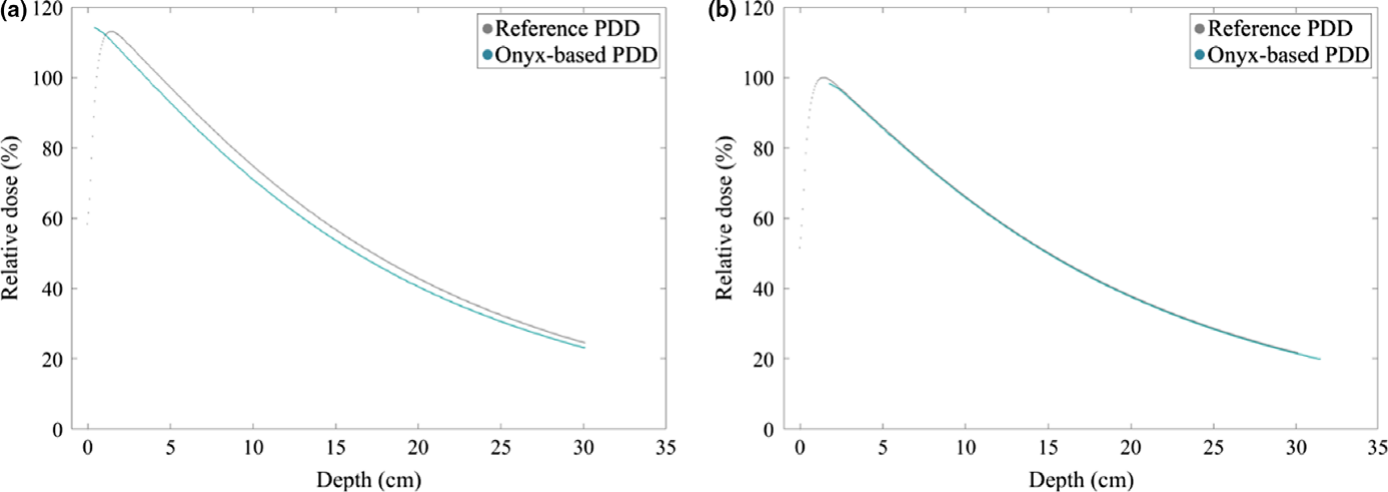
Percent depth dose curves for reference and Onyx‐based PDDs. (a) plots the raw data for the reference PDD (grey) and Onyx‐based PDD (blue). (b) plots the shifted (1.368 cm) and ISL corrected Onyx‐based PDD with the reference PDD.

The measured PDDs for the 6‐MV beam are shown in Fig. 4. The raw data for the reference PDD and Onyx‐based PDD are shown in Fig. 4(a). In Fig. 4(b) the shifted (1.368 cm) and ISL corrected Onyx‐based PDD is shown alongside the raw reference PDD.

The same measurements and analysis were performed on 6 and 10 FFF beams (not shown). The corrected Onyx‐based PDD shows good agreement with the reference PDD, indicating that a water equivalent estimate of the Onyx^™^ material is reasonable.

### 3.D Surface dose measurements

**Figure 5:**
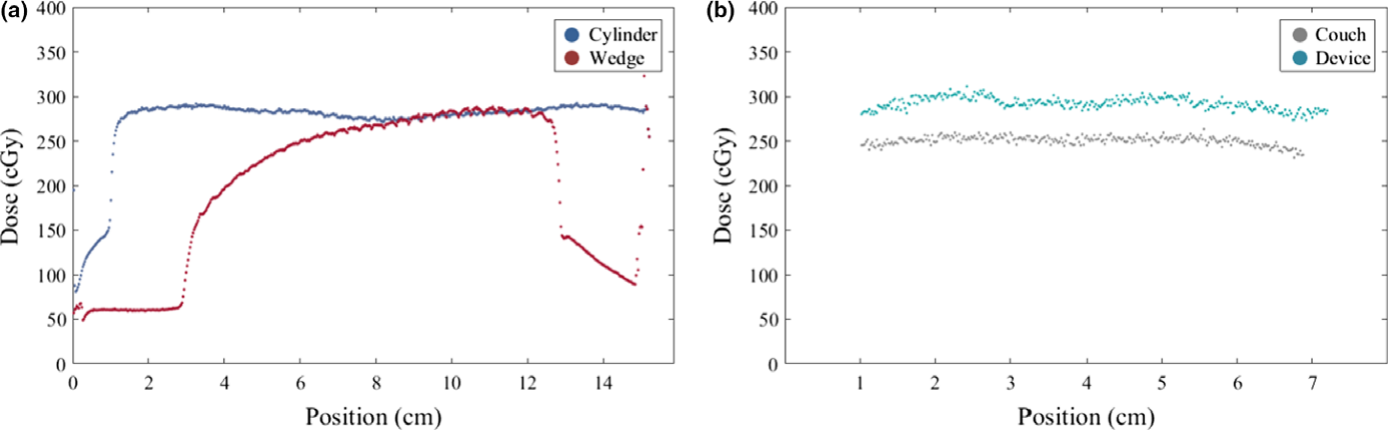
Surface dose measurements. (a) The in‐plane dose profiles of two representative objects, cylinder (blue) and wedge (red), demonstrate the surface dose effect of a patient‐specific device that may have varying thickness. (b) The in‐plane dose profiles for the couch surface (gray) and CSI immobilization board surface (blue) are displayed. The dose profile shown in (b) is limited to dose greater than 90% of the maximum dose.

A plot of the absolute dose measurements comparing the representative wedge to the representative cylinder are shown in Fig. 5(a). The wedge profile at maximum thickness (3 cm) is matched to the profile measured with the representative cylinder (also 3 cm). The infill pattern in the wedge surface dose measurement is evident when examining the repetitive undulating pattern in the thicker end of the wedge.

The absolute dose of the film measurements on the couch surface and CSI immobilization board surface are plotted in Fig. 5(b). The mean values of the dose profile for the couch dose and CSI dose are 251.2 and 292.5 cGy, respectively, which is a relative increase in dose of approximately 16%.

## 4 DISCUSSION

The customizability and simplicity of 3D printing aligns with radiotherapy aims of creating patient‐specific immobilization devices. With the recent increase in radiotherapy 3D‐printing literature, a likely trend towards greater integration of 3D printing in radiotherapy applications requires a comprehensive framework for clinical implementation of the devices. The presented framework builds on the existing scope of TG176,^10^ providing the additional measurements and considerations that are required for 3D‐printed immobilization devices compared to commercially manufactured radiotherapy devices. A summary of the proposed commissioning procedure, as illustrated in this manuscript, is as follows.

1 Estimate the water equivalent thickness of the print material.
2 Print two representative devices to be used as test objects:
  - A cylinder with diameter greater than the intended reference field and height that is representative of the intended device thickness.
  - A wedge with height equivalent to the height of the cylinder.
3 Perform attenuation measurements with the cylinder.
4 Perform surface dose measurements with the cylinder and wedge.
5 Acquire CT images of the attenuation experiment geometry and import into the treatment planning system to assess dose calculation accuracy. Compare the attenuation measurements in step (3) to the attenuation calculations in the treatment planning system.
6 Use wedge surface dose film measurements to assess the impact of shell thickness and/or infill pattern surface dose.
7 Acquire a reference and cylinder‐based PDDs. Cylinder‐based PDD can be shifted according to inverse square law and water equivalent thickness and assessed for agreement. Additionally, the wedge surface dose film measurements can be compared with point estimates from the reference PDD as shifted by the appropriate water equivalent thickness.

These measurements are intended for commissioning the 3D‐printed material for use with the treatment planning system. In addition to the presented measurements, a separate full clinical commissioning of the 3D‐printed device is required as part of *end‐to‐end* testing of implementing a new immobilization method that is specific to each treatment technique where the device could be used.

The measurement of attenuation and surface dose performed in this work are consistent with TG176. The TG176 report recommends the use of water equivalent thickness values to estimate surface dose using PDD data, and this work expands on that for unknown device geometry and materials. The measurement of the attenuation for the representative cylinder informs the clinician of the agreement with the treatment planning system in a simple scenario, in contrast to the comparison made when using the clinical device. If only the intended clinical device is used and there is a clinically relevant discrepancy in attenuation, it may be challenging to determine the cause due to the complex nature of these objects. For the CSI immobilization board, the absolute difference in attenuation and the clinical impact will be highly dependent on the intended treatment technique and site, which requires a clinical judgment by the commissioning team. In the demonstrated material, the differences in measurement and calculation were small. Ultimately, the discrepancies between measured and calculated attenuation demonstrate the treatment planning system's dose calculation accuracy in the context of the proprietary material.

A unique aspect of 3D printing is that objects are typically manufactured with a thin shell as well as an infill density and pattern that is user defined (honeycomb, pillars, etc.).^7^, ^12^ Although the measurement of density may appear to be straightforward, the relationship between shell thickness and infill density as they contribute to physical density vary depending on the printed device. The precision of the density in a printed device and its effect on dosimetry requires investigation before clinical implementation. The purpose of the wedge measurements is to ensure the water equivalent thickness is valid for the range of densities and thicknesses to be used.

The dosimetric behaviour of the infill pattern was characterized for specific materials in application as bolus by Ricotti et al.^3^ and found that for infill percentages greater than 20%, the material could be approximated as homogeneous. However, this relationship is not known for other 3D‐printed materials, which highlights the importance of surface dose measurements that may yield a dosimetric pattern that is reflective of the print pattern. In Fig. 5(a), the infill pattern is evident as the attenuation of the incident photons became greater at the thicker end of the wedge. This effect, if any, should be known to the clinician and any judgement of its clinical significance can be made prior to clinical implementation. The measured PDDs can also be used to confirm that the material is appropriately estimated with a water equivalent thickness. Though PDDs are relatively insensitive to a change in energy spectrum, agreement in the reference and material‐based PDDs provide the clinician confidence that the dose calculation algorithm will accurately compute dose in the material. Methodologies for estimating electron density from CT number have been previously explored.^13^, ^14^ Notably, Michiels et al.^2^ reported on the use of dual‐energy CT to estimate the effective atomic number of a 3D‐printed material. However, this is not practical for routine clinical implementation but could be used as an investigational tool. The surface dose and PDD measurements are designed to provide further evidence that the material properties are well understood in the radiotherapy context.

This work outlines the characterization framework required for use of 3D‐printed materials as an immobilization device. For use as bolus, these dosimetric measurements are useful but the additional requirement to assess the intended clinical device's reproducibility in patient setup should be evaluated. Often, the rigidity of the 3D‐printed materials may limit the application to malleable bolus devices. Furthermore, the case study results presented in this work do not assess the consistency in print, which could be important if devices are intended for disposable use. In that scenario, multiple test objects and a relevant subset of these recommended dosimetric experiments could be repeated to understand the variation observed across devices.

## 5 CONCLUSION

A framework is presented for dosimetric characterization of 3D‐printed patient support and immobilization devices. The study performs measurements on representative test objects and uses a case study of a 3D‐printed CSI immobilization board to outline requirements for clinical commissioning of the 3D‐printed material. Recommendations are made to clearly define a series of steps that can be implemented by a user with access to generic 3D printers and materials.

## CONFLICTS OF INTEREST

The authors recognize a conflict of interest in that the 3D‐printed material (Onyx^™^) provided for this study was donated by MarkForged.
